# Antibiotic Use Among Hospitalized Patients in Africa: A Systematic Review of Point Prevalence Studies

**DOI:** 10.1007/s40615-023-01610-9

**Published:** 2023-05-08

**Authors:** Usman Abubakar, Muhammad Salman

**Affiliations:** 1 Department of Clinical Pharmacy and Practice, College of Pharmacy, QU Health, Qatar University, Doha, Qatar; 2 Institute of Pharmacy, Faculty of Pharmaceutical and Allied Health Sciences, Lahore College for Women University, Lahore, Pakistan

**Keywords:** Point prevalence, Africa, Antibiotic prescribing, Antibiotic use, Hospitalized patients, Systematic review

## Abstract

**Background:**

There is paucity of data describing the rate and quality indices of antibiotics used among hospitalized patients at continental level in Africa. This systematic review evaluated the pooled prevalence, indications, and types of antibiotics used in hospitals across Africa.

**Methods:**

Three electronic databases, PubMed, Scopus, and African Journals Online (AJOL), were searched using search terms. Point prevalence studies of antibiotic use in inpatient settings published in English language from January 2010 to November 2022 were considered for selection. Additional articles were identified by checking the reference list of selected articles.

**Results:**

Of the 7254 articles identified from the databases, 28 eligible articles involving 28 studies were selected. Most of the studies were from Nigeria (*n* = 9), Ghana (*n* = 6), and Kenya (*n* = 4). Overall, the prevalence of antibiotic use among hospitalized patients ranged from 27.6 to 83.5% with higher prevalence in West Africa (51.4–83.5%) and North Africa (79.1%) compared to East Africa (27.6–73.7%) and South Africa (33.6–49.7%). The ICU (64.4–100%; *n* = 9 studies) and the pediatric medical ward (10.6–94.6%; *n* = 13 studies) had the highest prevalence of antibiotic use. Community-acquired infections (27.7–61.0%; *n* = 19 studies) and surgical antibiotic prophylaxis (SAP) (14.6–45.3%; *n* = 17 studies) were the most common indications for antibiotic use. The duration of SAP was more than 1 day in 66.7 to 100% of the cases. The most commonly prescribed antibiotics included ceftriaxone (7.4–51.7%; *n* = 14 studies), metronidazole (14.6–44.8%; *n* = 12 studies), gentamicin (*n* = 8 studies; range: 6.6–22.3%), and ampicillin (*n* = 6 studies; range: 6.0–29.2%). The access, watch, and reserved group of antibiotics accounted for 46.3–97.9%, 1.8–53.5%, and 0.0–5.0% of antibiotic prescriptions, respectively. The documentation of the reason for antibiotic prescription and date for stop/review ranged from 37.3 to 100% and 19.6 to 100%, respectively.

**Conclusion:**

The point prevalence of antibiotic use among hospitalized patients in Africa is relatively high and varied between the regions in the continent. The prevalence was higher in the ICU and pediatric medical ward compared to the other wards. Antibiotics were most commonly prescribed for community-acquired infections and for SAP with ceftriaxone, metronidazole, and gentamicin being the most common antibiotics prescribed. Antibiotic stewardship is recommended to address excessive use of SAP and to reduce high rate of antibiotic prescribing in the ICU and pediatric ward.

## Background

Antimicrobial resistance remains a major public health challenge in the twenty-first century [1–3]. It threatens the use of antibiotics for the prevention of infections due to surgery, dialysis, and chemotherapy [4]. Infections caused by multidrug-resistant pathogens are associated with high mortality rate [5, 6] and significant morbidity and healthcare costs [3]. Infections caused by resistant pathogens, especially the multidrug-resistant pathogens, are difficult to treat due to limited number of effective antibiotics [6, 7]. Infections due to antibiotic-resistant pathogens cause an estimated 700,000 deaths per year, and this was estimated to increase to about 10 million deaths per year by the year 2050 [8]. This calls for interventions to reduce the burden of antibiotic resistance in healthcare system. Inappropriate use of antibiotics contributes to the emergence and transmission of antibiotic-resistant pathogens [9]. Evidence has shown that about 20–50% of antibiotic prescriptions are inappropriate, and this increases the risk of antibiotic resistance [10]. Antimicrobial stewardship program is used as a strategy to tackle inappropriate antibiotic prescription in healthcare facilities and prevent antibiotic resistance [11]. Evidence has demonstrated the effectiveness of antimicrobial stewardship in improving antibiotic prescribing practices among prescribers and improving clinical and microbial outcomes [12, 13]. In addition, antimicrobial stewardship has been shown to reduce healthcare cost among patients [14].

Evaluation of antibiotic prescribing pattern among patients in healthcare facilities is used to identify antimicrobial stewardship opportunities to improve appropriate use of antibiotics [15]. Point prevalence studies have been found to be valid and reliable in measuring antibiotic use among hospitalized patients [16]. Available evidence has shown that about 30% and 50% of hospitalized patients in Europe and the USA use at least one antibiotics per day [17, 18]. In Africa, several point prevalence studies have reported high rate of antibiotic use among hospitalized patients and inappropriate use of antibiotics in healthcare facilities [19–22]. However, there is limited data to describe the point prevalence of antibiotic use among hospitalized patients in Africa at a regional level. Understanding the epidemiology of antibiotic use among hospitalized patients and the quality of antibiotic prescribing is important to design effective antimicrobial stewardship interventions to promote rational use of antibiotics and improve clinical outcomes among patients. The objective of this study is to evaluate the prevalence of antibiotic prescribing among hospitalized patients, the prevalence of antibiotic use in different hospital ward/unit, and the quality indicators of antibiotic prescriptions in healthcare facilities across Africa.

## Methods

### Study Design

This systematic review of antibiotic use among hospitalized patients in Africa was conducted in accordance with the Preferred Reporting Items for Systematic review and Meta-Analysis (PRISMA) statement 2020 [23].

### Eligibility Criteria

#### Inclusion Criteria

1. Point prevalence studies conducted among hospitalized patients in acute care settings in Africa
2. Studies published between January 2010 and 3 November 2022. The review was limited to studies published from January 2010 in order to provide estimates of the outcomes based on recent studies. In addition, most point prevalence surveys conducted among hospitalized patients in Africa were published from 2010 onwards.
3. Studies conducted in all age groups and all inpatient settings
4. Studies that were published in English language and available as free full text

#### Exclusion Criteria

1. Longitudinal studies that accessed antibiotic use among hospitalized patients
2. Point prevalence studies that evaluated antibiotic use in outpatient settings. This is because the current review was focused on antibiotic use among hospitalized patients only.
3. Point prevalence survey of antibiotic use in a specific patient population such as COVID-19 patients only was excluded.
4. Studies that described antibiotic consumption in defined daily doses without the rate of antibiotic use
5. Previous systematic reviews and meta-analyses, editorials, letters to editors, commentaries, and unpublished articles of antibiotic use

### Information Sources

Three electronic databases including PubMed, Scopus, and African Journals Online (AJOL) were searched to identify eligible articles. The search was conducted using the search terms described under search strategy. Google Scholar search was also conducted to find eligible articles. Additional search was conducted by checking the reference lists of selected articles.

### Search Strategy

The search terms used include “point-prevalence study,” “antibiotic use,” and “Africa” along with their synonyms. The terms were combined using Boolean operators. The search terms used for the electronic search are as follows: Antibiotic use OR Antibiotic prescribing OR Antimicrobial use OR antimicrobial prescribing AND hospitalized patients OR acute care patients AND Africa AND point prevalence survey OR point-prevalence study.

### Selection Process

The results of the electronic search were combined and checked for the removal of duplicate articles. Screening of the titles and abstracts of non-duplicate articles was conducted to identify potentially eligible articles. Ineligible articles at this stage were excluded. The full text of the articles that fulfilled eligibility criteria were assessed for final selection and for data collection.

### Data Collection Process

The selected articles were assessed for quality and reviewed for data collection using a predesigned form. Data was extracted by one independent reviewer (UA), and the extracted information was checked by the second reviewer. Consensus was used to address any disagreements between the reviewers.

### Data Items

The data items collected from the selected articles include first author’s name and year of publication, country, study setting/number of center(s), study design, study duration, number of patients involved, PPS protocol used (ECDC, CDC, or as defined by the authors), overall prevalence of antibiotic use, prevalence of antibiotic use in different wards, indications for antibiotic use, types of antibiotic, and quality indicators such as duration of surgical antimicrobial prophylaxis, redundant antibiotic use, and documentation of reason for antibiotic use.

### Quality Assessment

Quality assessment for the selected articles was performed using the Newcastle–Ottawa scale (NOS) [24]. The NOS consists of three sections including selection, comparability, and outcomes. Quality assessment was conducted by an independent reviewer (MS), and the result was randomly checked by a second reviewer (UA). Disagreements between the reviewers were resolved through consensus.

## Results

### Study Selection

**Fig. 1:**
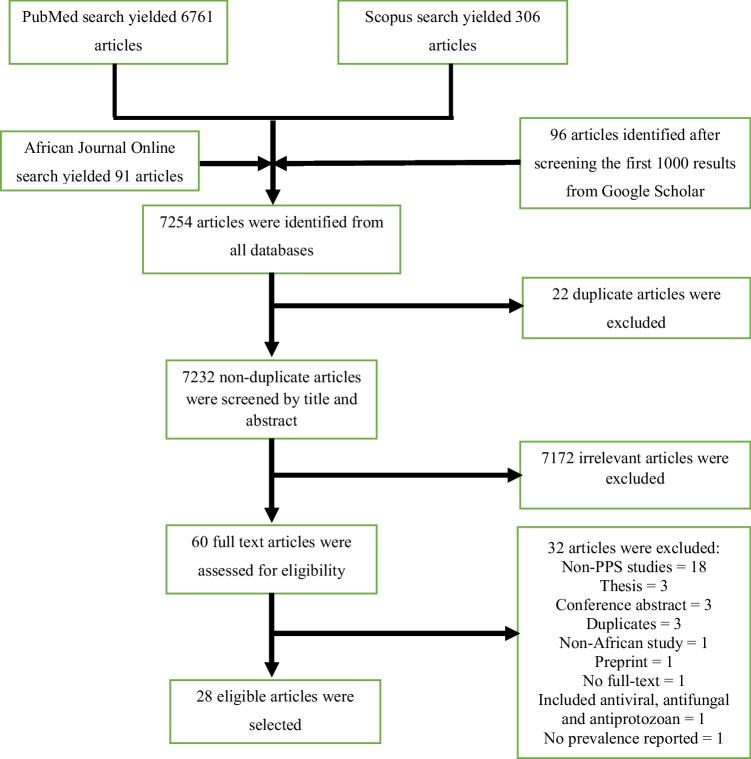
Flow chart of article screening and selection

The search conducted on PubMed, Scopus, and African Journal Online databases yielded 6761, 306, and 91 articles, respectively. A total of 96 articles were identified after screening the first 1000 results from Google Scholar. Overall, 7254 articles were retrieved from the databases after the search. Of the 7254 articles, 22 duplicate articles were identified and removed. The title and abstract of the remaining articles were screened to remove articles that were not relevant to this systematic review and meta-analysis. The full text of 60 articles was assessed for inclusion based on the eligibility criteria, and 28 articles from 28 studies were selected. Figure 1 illustrates the procedure used during the screening and selection process.

### Characteristics of Selected Studies

Most of the selected studies were from Nigeria (*n* = 9), Ghana (*n* = 6), Kenya (*n* = 4), Tanzania (*n* = 2), and South Africa (*n* = 2). Most of the studies (*n* = 17) involved multiple centers and 9 and 2 studies conducted in single and two centers, respectively. Majority of the studies were conducted before COVID-19 pandemic with 10 studies conducted in 2019 and four each in 2016 and 2017. There were two studies conducted in 2021 and one study in 2020. The number of patients involved in the selected studies ranged from 113 to 4407 patients. Most studies (*n* = 24) included patients from different wards while two studies each involved only surgical and pediatric population. Table 1 summarizes the characteristics of the selected studies.

**Table 1:** Characteristics of the studies included in this review

S. no.	Author and year	Country	Study setting/no. of centers	Protocol used	Period of the study	Number of patients	Prevalence of antibiotic use (%)	Prevalence of antibiotic use in different wards (%)	Indication for antibiotic use (%)	Classes of antibiotics used (%)	Types of antibiotic used (%)	AWaRe classification (%)
1	Usman (2020) [22]	Nigeria	Hospital-wide/multicenter	ECDC protocol	April–May 2019	321	257/321 (80.1%)	Pediatric medical: 94.6 Neonatal: 92.9 Medical:73.6 Surgical: 78.3 OBG: 72.9 Pediatric surgical: 90.0	CI: 38.7 HI: 16.3 MP: 14.9 SAP: 22.5 Unknown: 7.6	Nitroimidazole: 28.5 Third-generation cephalosporin: 18.9 Fluoroquinolone: 13.6 BLBLI: 10.5 Aminoglycoside: 8.5	Metronidazole: 30.5 Ciprofloxacin: 17.1 Ceftriaxone: 16.8 Augmentin: 12.5 Gentamicin: 11.8	NA
2	Aboderin et al. (2021) [21]	Nigeria	Hospital-wide/multicenter	WHO protocol	10–27 June 2019	321	246/321 (76.6%)	Medical: 19.5 Surgical: 22.9 Pediatric: 10.6 Ortho: 9.2 NNW/NICU: 13.7 Gynecology: 6.7 Postnatal: 11.9	CI: 29.2 HI: 8.8 SAP: 36.9 MP: 11.2 Others: 13.8	NA	Metronidazole: 25.2 Cefuroxime: 18.4% Ceftriaxone: 13.7 Ciprofloxacin: 10.6 Gentamicin: 10.5	Access: 46.3 Watch: 53.5 Reserve: 0.2
3	Afriyie et al. (2020) [25]	Ghana	Hospital-wide/bicentric	Global PPS protocol	May 2019	NA	GPH: 65% KMH: 82%	Medical: 56.6–73.7 Surgical: 46.7–50.0 Pediatric medical: 77.8–100 Pediatric surgical: 100 NNW: 100	CI: 79.5–100 HI: 0–20.5 SAP: 59.1–72. 2 MP: 27.8–40.9	NA	NA	NA
4	Ahoyo et al. (2012) [26]	Benin Republic	Hospital-wide/multicenter	HELICS protocol	10–26 October 2012	3130	2023/3130 (64.6%)	NA	NA	Beta-lactam: 86.9% Cephalosporin: 17.4% Quinolone: 8.5% Imidazole: 7.5 Aminoglycoside: 6.0%	NA	NA
5	Amponsah et al. (2021) [27]	Ghana	Hospital-wide/multicenter	WHO protocol	November–December 2019	190	115/190 (60.5%)	NA	CI: 36.5 HI: 15.7 SAP: 26.1 MP: 13.9 Others: 7.8	Penicillin: 48.7% Cephalosporin: 23.5 Quinolone: 17.4 Lincosamide: 4.4 Aminoglycoside: 2.6	Amoxicillin: 36.5 Ciprofloxacin: 17.4 Ceftriaxone: 11.3 Cefuroxime: 9.6 Ampicillin: 7.8	NA
6	Ashour et al. (2022) [20]	Egypt	Hospital-wide/single center	ESAC protocol	16–29 July 2019	379	300/379 (79.1%)	NA	CI: 28.7 HI: 9.0 SAP: 45.3 MP: 17.0	BLBLI: 43.3 Third-generation cephalosporin: 29.0 Quinolone: 8.7 First-generation cephalosporin: 6.3 Fourth-generation cephalosporin: 4.0	NA	Aware: 52 Watch: 43 Reserve: 5
7	Bediako-Bowan et al. (2019) [19]	Ghana	Surgical unit/multicenter	ECDC protocol	September–December 2016	540	382/540 (70.7%)	NA	CI: 174/382 (45.5%). HI: 50/382 (13.1%) MP: 23/ (6.0%) SAP: 121 (31.7%) Unknown: 14	Nitroimidazole: 25.6 Second- and third-generation cephalosporin: 20.0 BLBLI: 16.7 Quinolone: 12.3 Lincosamide: 10.2	NA	NA
8	Bunduki et al. (2021) [28]	Malawi	Surgery department/single center	Adapted ECDC protocol	9 June 2020	113	29/113 (27.6%)	NA	Prophylaxis: 10.3% Treatment: 48.3%	3^rd^ gen cephalosporin: 51.7% Metronidazole: 44.8 Amoxicillin: 24.1 Doxycycline: 13.8 Ciprofloxacin: 13.8	Ceftriaxone: 51.7 Metronidazole: 44.8 Amoxicillin: 24.1 Doxycycline: 13.8 Ciprofloxacin: 13.8	NA
9	Nsofor et al. (2016) [29]	Nigeria	Hospital-wide/multicenter	ESAC protocol	NA	1585	886/1585 (55.9%)	NA	NA	NA	Chloramphenicol: 33.3 Tetracycline: 33.2 Ampicillin: 29.3 Amoxicillin: 28.9 Erythromycin: 26.4	NA
10	Fentie et al. (2022) [30]	Ethiopia	Hospital-wide/multicenter	WHO PPS protocol	January 2021	1820	1162/1820 (63.8%)	Surgical: 66.4 Medical: 58.5 OBG: 50.8 NICU: 76.1 Pediatric medical: 76.7 ICU: 86 Pediatric surgical: 73.2 PICU: 69.2	CI: 33.8 HI: 40.3 SAP: 18.3 MP: 7.2 Unknown: 0.3	NA	NA	NA
11	Horumpende et al. (2020) [31]	Tanzania	Hospital-wide/multicenter	ECDC protocol	November–December 2016	399	176/399 (44%)	Medical: 35% Surgical: 40%	CI: 42.0% HI: 10 SAP: 30% MP: 0.5 Unknown: 11%	Ceftriaxone: 28.5 Metronidazole: 23.9 Penicillins: 26.9 Aminoglycoside: 6.6 Cotrimoxazole: 3.9%	Ceftriaxone: 28.5 Metronidazole: 23.9 Ampiclox:8.5 ampicillin: 7% Gentamicin: 6.6	NA
12	Kamita et al. (2022) [32]	Kenya	Hospital-wide/single center	Adapted global PPS protocol	July 2021	308	191/308 (62.0%)	ICU: 100 Pediatric: 94.1 Medical: 69.2 Gynecology: 65.6 Surgical: 64.1 Postnatal: 56.3 Neonatal: 45.5	CI: 34.5% HI: 1.2 SAP: 14.6 MP: 12.3 Unknown: 36.3 Others: 1.2	NA	NA	Access: 57 Watch: 42
13	Fowotade et al. (2020) [33]	Nigeria	Hospital-wide/single center	Global PPS protocol	December 2017	451	426/451 (60.5%)	NA	CI: 119/430 (27.7%) HI: 53/430 (12.3%) SAP: 176/430 (40.9%) MP: 75/43 (17.4%) Unknown: 7/430	Cephalosporin: 30% Metronidazole: 18 BLBLI: 16 Aminoglycoside: 11 Quinolones: 15	Ceftriaxone: 15.6% Metronidazole: 14.6 Augmentin: 11.6 Ciprofloxacin: 9.1 Gentamicin: 8.6%	NA
14	Kiggundu et al. (2022) [34]	Uganda	Hospital-wide/multicenter	WHO PPS protocol	December 2020–April 2021	1077 patients	73.7%	NA	CI: 41.6% HI: 6.3% SAP: 23.0% MP: 29.1	NA	Ceftriaxone: 37% Metronidazole: 27% Gentamicin: 7% Ampicillin: 6% Ampiclox: 6%	Access: 47.2 Watch: 44.1 Unclassified: 9.0 Reserve: 0.0
15	Labi et al. (2018) [35]	Ghana	Hospital-wide/single center	ESAC protocol	February–March 2016	677 patients	348/677 (51.4%)	OBG: 36% Pediatric surgical: 90.9% Gynecology: 44.7 Medical: 50.0 Surgery: 56.9 Pediatric: 69.4	CI: 40.1% HI: 21.0% SAP: 33.6 MP: 5.4	Penicillin: 24.9% Nitroimidazole: 17.5% Third-generation cephalosporin: 13.8 Second-generation cephalosporin: 10.0 Aminoglycoside: 8.8	Metronidazole: 17.5 Augmentin: 13.4% Ceftriaxone: 12.1% Cefuroxime: 10.0% Cloxacillin: 8.5%	NA
16	Labi et al. (2021) [36]	Ghana	Hospital-wide/multicenter	Global PPS	September–December 2019	2897 patients	1562/2897 (53.9%)	Medical: 51.3 Surgical: 50.1 IUC: 89.3 Neo medical: 63.1 NICU: 53.1 Pediatric medical: 73.2 Pediatric surgical: 56.7 PICU: 45.8	SAP: 26.1% MP: 8.0% Unknown: 13.7%	NA	Metronidazole: 20.6% Cefuroxime: 12.9% Ceftriaxone: 11.8% Amoxicillin/clavulanic acid: 8.8% Ciprofloxacin: 7.8%	NA
17	Labi et al. (2018) [37]	Ghana	Pediatric units/multicenter	Adapted ECDC protocol	September–December 2016	716 patients	506/716 (70.6%)	NA	CI: 61.0% HI: 10.3 Prophylaxis: 23.7 Unknown: 4.8%	Third-generation cephalosporin: 18.5% Aminoglycoside: 17.9% Second-generation cephalosporin: 12.4 Beta-lactam-resistant penicillin: 10.0 Nitroimidazole: 9.9	NA	NA
18	Momanyi et al. (2019) [38]	Kenya	Hospital-wide/single center	Global PPS	April 2017	179 patients	54.7%	ICU: 100% Neonatal: 93.7 Pediatric medical: 84.2% Medical: 61.5 Surgical: 57.3 OBG: 20.8	CI: 54.2 HI: 2.8 SAP: 26.3 MP: 15.1	Penicillin: 46.9 Cephalosporins: 44.7 Aminoglycosides: 26.3	Ceftriaxone: 39.7% Benzylpenicillin: 29.0% Metronidazole: 25.1% Gentamicin: 22.3% Flucloxacillin:11.2	NA
19	Nnadozie et al. (2021) [39]	Nigeria	Hospital-wide/single center	Global PPS	May 2019	127 patients	106/127 (83.5%)	NA	CI: 65 HI: 5.3 Prophylaxis: 29.4 Unknown: 0.3	NA	Ceftriaxone: 25.7 Tinidazole: 21.9 Metronidazole: 14.6 Cefuroxime: 7.0 Levofloxacin: 5.6	NA
20	Oduyebo et al. (2017) [40]	Nigeria	Hospital-wide/multicenter	NA	April–June 2015	828 patients	577/828 (69.7%)	ICU: 88.9 Pediatric medical: 84.6 NICU:76.8 Pediatric. surgical: 70.7 Surgical: 67.7 Medical:63.3 Neonatal medical: 60.6 Hematology/oncology: 25.0	CI: 468/1022 (45.79%) HI: 55/1022 (5.38%) SAP: 277/1022 (27.1%) MP: 120 (11.7) Unknown: 102/1022	Third-generation cephalosporin: 21.4% Metronidazole: 18.0 Quinolones: 14.1	NA	NA
21	Ogunleye et al. (2022) [41]	Nigeria	Hospital-wide/bicentric	Adapted ECDC and global PPS protocol	November 2019	491 patients	398/494 (80.6%)	NA	CI: 41.5 HI: 5.7	Cephalosporin: 43.5% Nitroimidazole: 28.8% Penicillins: 11.0% Quinolones: 5.8% Aminoglycoside: 4.4%	Ceftriaxone: 26.0% Metronidazole: 28.8% Augmentin: 8.9% Cefuroxime: 5.4% Levofloxacin: 3.5%	NA
22	Okoth et al. (2018) [42]	Kenya	Hospital-wide/single center	Global PPS	5–12 June 2017	269 patients	182/269 (67.7%)	Postnatal: 92.5 Neonatal: 83.3 ICU: 66.7 Medical: 64.3 Gynecology: 64.3 Surgical:61.9 Pediatrics: 58.7	CI: 28% HI: 13 SAP: 22% MP: 29% Others: 6% Unknown: 2	Third-gen cephalosporin: 55% Imidazole: 41.8 Broad spectrum penicillin: 41.8% Aminoglycoside: 7.1%	NA	NA
23	Omulo et al. (2022) [43]	Kenya	Hospital-wide/multicenter	WHO protocol	September 2017 and March–April 2018	1071 patients	489/1071 (46.0%)	ICU: 82% Medical: 38.0% OBG: 48% Pediatric: 59% Surgical: 40%	NA	NA	NA	NA
24	Seni et al. (2020) [44]	Tanzania	Hospital-wide/multicenter	WHO protocol	December 2019	948 patients	591/948 (62.3%)	Medical: 47.9 Surgical: 82.4 Pediatric: 84.3 ICU:64.4	CI: 39.8% HI: 5.4 SAP: 28.8% MP:22.8%	NA	Ceftriaxone: 30.9% Metronidazole: 22.9% Ampicillin–cloxacillin: 17.0% Gentamicin: 11.0% Ampicillin: 6.9%	Access: 97.9 Watch: 1.8 Reserve: 0.3
25	Skosana et al. (2021) [45]	South Africa	Hospital-wide/multicenter	ECDC and global PPS	April–August 2018	4407 patients	1479/4407 (33.6%)	NA	NA	NA	NA	Access: 54.6 Watch: 30.2 Reserve: 1.9 Unclassified: 13.3
26	Skosana et al. (2021) [46]	South Africa	Pediatric/multicenter	ECDC protocol	April–August 2018	1261 patients	627/1261 (49.7%)	Pediatric medical: 74.7 Pediatric surgical: 9.6 PICU: 15.7	Prophylaxis: 16.4 Treatment: 83.6%	NA	Ampicillin: 16.4% Gentamicin: 10.0% Amoxicillin/enzyme inhibitor: 9.6% Ceftriaxone: 7.4% Amikacin: 6.3%	Access: 55.9 Watch: 27.8 Reserve: 3.1 Unclassified: 13.2
27	Umeokonkwo et al. (2019) [47]	Nigeria	Hospital-wide/single center	Global PPS protocol	October–November 2017	220 patients	172/220 (78.2%)	ICU: 100 Adult surgical: 82.9 Pediatric medical: 82.9 Neonatal medical: 77.8 Pediatric surgical: 75.0 Adult medical: 70.7	CI: 45.5 HI: 6.0 SAP: 44.0 MP: 2.9 Unknown: 1.6	Metronidazole: 33.9 Third-generation cephalosporin: 37.5% Second-generation cephalosporin: 7.7	NA	NA
28	Manga et al. (2021) [48]	Nigeria	Hospital-wide/single center	Global PPS protocol	April 2019	326 patients	70.6% adult 73.4% pediatric	Medical: 70.6% Pediatric: 73.4%	NA	Cephalosporins:29.2% Penicillins: 22. 8% Fluoroquinolones: 12.4 Aminoglycosides: 9.1 Macrolides: 3.4	NA	NA

*NA*, not applicable; *ECDC*, European Centre for Disease Prevention and Control; *WHO*, World Health Organization; *PPS*, point prevalence study; *HELICS*, Hospitals in Europe Link for Infection Control through Surveillance; *ESAC*: European Surveillance of Antimicrobial Consumption; *CI*, community-acquired infections; *HI*, hospital-acquired infections; *SAP*, surgical antimicrobial prophylaxis; *MP*, medical prophylaxis; *NICU*, neonatal intensive care unit; *NNW*, neonatal ward; *PICU*, pediatric intensive care unit; *ICU*, intensive care unit; *OBG*, obstetrics and gynecology; *BLBLI*, beta-lactam beta-lactamase inhibitor combination

### Quality Assessment of Selected Studies

Almost all the studies included a population that is either truly or somewhat representative of the target population. Similarly, almost all the included studies had a sample size that is satisfactory and justified. The quality score among the included studies ranged from 4 to 9 with 23 studies (82.1%) scoring >7 points. Overall, 24 studies (85.7%) were found to have good quality while 3 studies had fair quality. One study was adjudged to have poor quality. Table 2 shows the quality assessment results of the studies included in this review.

**Table 2:** Quality assessment of the studies included in the review

Author name and year	Selection				Comparability	Outcomes		Quality score	Quality scale
	Representatives of sample	Sample size	Non-respondents	Ascertainment of exposure	Based on design and analysis	Assessment of outcomes	Statistical test		
Usman (2020) [22]	*	*	NA	**	--	**	*	7	Good
Aboderin et al. (2021) [21]	*	*	NA	**	--	**	*	7	Good
Afriyie et al. (2020) [25]	*	*	NA	**	--	**	--	6	Fair
Ahoyo et al. (2012) [26]	*	*	NA	**	--	**	*	7	Good
Amponsah et al. (2021) [27]	*	*	NA	**	**	**	*	9	Good
Ashour et al., 2022 [20]	*	*	NA	**	--	**	*	7	Good
Bediako-Bowan et al. (2019) [19]	*	*	NA	**	*	**	*	8	Good
Bunduki et al. (2021) [28]	*	*	NA	**	--	**	*	7	Good
Nsofor et al. (2016) [29]	--	--	NA	**	--	**	--	4	Poor
Fentie et al. (2022) [30]	*	*	NA	**	**	**	*	9	Good
Horumpende et al. (2020) [31]	*	*	NA	**	--	**	*	7	Good
Kamita et al. (2022) [32]	*	*	NA	**	--	**	*	7	Good
Fowotade et al. (2020) [33]	*	*	NA	**	--	**	--	6	Fair
Kiggundu et al. (2022) [34]	*	*	NA	**	**	**	*	9	Good
Labi et al. (2018) [35]	*	*	NA	**	--	**	*	7	Good
Labi et al. (2021) [36]	*	*	NA	**	--	**	*	7	Good
Labi et al. (2018) [37]	*	*	NA	**	--	**	*	7	Good
Momanyi et al. (2019) [38]	*	*	NA	**	--	**	*	7	Good
Nnadozie et al. (2021) [39]	*	*	NA	**	--	**	*	7	Good
Oduyebo et al. (2017) [40]	*	*	NA	*	--	**	--	5	Good
Ogunleye et al. (2022) [41]	*	*	NA	**	--	**	*	7	Good
Okoth et al. (2018) [42]	*	*	NA	**	--	**	*	7	Good
Omulo et al. (2022) [43]	*	*	NA	**	--	**	*	7	Good
Seni et al. (2020) [44]	*	*	NA	**	*	**	*	8	Good
Skosana et al. (2021) [45]	*	*	NA	**	--	**	*	7	Good
Skosana et al. (2021) [46]	*	*	NA	**	--	**	*	7	Good
Umeokonkwo et al. (2019) [47]	*	*	NA	**	--	**	--	6	Fair
Manga et al. (2021) [48]	*	*	NA	**	--	**	*	7	Good

*NA*, not applicable

### Qualitative Summary of Results

#### Overall Prevalence and Prevalence of Antibiotic Use in Different Wards/Units Among Hospitalized Patients in Africa

Overall, the prevalence of antibiotic use among hospitalized patients in Africa ranged from 27.6 to 83.5% [28, 39]. The prevalence of antibiotic use was higher in West Africa (ranged from 51.4 to 83.5%) [19, 21, 22, 25–27, 29, 33, 35–37, 39–41, 47, 48], followed by North Africa (79.1%) [20], East Africa (ranged from 27.6 to 73.7%) [28, 30–32, 34, 38, 42–44], and South Africa (ranged from 33.6 to 49.7%) [45, 46]. The highest prevalence of antibiotic use was found in Nigeria (83.5%) [39], Ghana (82%) [25], Egypt (79.1%) [20], and Uganda (73.7%) [34]. The lowest rate of antibiotic use was observed in Malawi (27.6%) [28], followed by South Africa (33.6%) [45] and Tanzania (44.0%) [31]. Table 1 summarizes the prevalence and types of HAIs reported in the selected studies. The prevalence of antibiotic use was higher among patients admitted to the ICU (64.4–100%; *n* = 9 studies) [30, 32, 36, 38, 40, 42–44, 47], followed by pediatric medical (10.6–94.6%; *n* = 13 studies) [21, 22, 30, 32, 35, 36, 38, 40, 42–44, 47, 48], neonatal (45.5–93.7%; *n* = 7 studies) [22, 32, 36, 38, 40, 42, 47], pediatric surgery (56.7–90.7%; *n* = 6 studies) [22, 30, 35, 36, 40, 47], and adult surgical (22.9–82.9%, *n* = 12 studies) [21, 22, 30, 32, 35, 36, 38, 40, 42–44, 47] ward. The rate of antibiotic use among patients hospitalized on other wards includes neonatal ICU (53.1–76.8%; *n* = 3 studies) [30, 36, 40], adult medical (19.5–73.6%; *n* = 13 studies) [21, 22, 30, 32, 35, 36, 38, 40, 42–44, 47, 48], and OBG/postnatal (6.7–92.5%; *n* = 8 studies) [21, 22, 30, 32, 35, 38, 42, 43] wards.

#### Indication for Antibiotic Use and the Routes of Administration

The indications for antibiotic use among hospitalized patients varied between the studies. Community-acquired infections were the most common indication for antibiotic use and ranged from 27.7 to 61.0% (*n* = 19 studies) [19–22, 27, 30–35, 38–42, 44, 47], followed by surgical antimicrobial prophylaxis (14.6–45.3%; *n* = 17 studies) [19–22, 27, 30–36, 38, 40, 42, 44, 47]. Hospital-acquired infections (1.2–40.3%; *n* = 19 studies) [19–22, 27, 30–35, 38–42, 44, 47] and medical prophylaxis (0.5–29.1%; *n* = 17 studies) [19–22, 27, 30–36, 38, 40, 42, 44, 47] were the other indications for antibiotic use in African settings. Both oral and parenteral antibiotics are used among hospitalized patients in Africa. The parenteral antibiotics were the most commonly used and accounted for 54.0–98.6% of all antibiotics (*n* = 18 studies) [19–22, 30, 32–37, 39, 41, 42, 45–48] while oral antibiotics accounted for 11.0–46.0% (*n* = 11 studies) [19, 21, 22, 32, 34, 37, 39, 42, 45–47].

#### Antibiotic Used Among Hospitalized Patients

A total of 15 studies reported top five most commonly prescribed antibiotics in inpatient settings in Africa. Based on the results, ceftriaxone (*n* = 14 studies) and metronidazole (*n* = 12 studies) were the most commonly used antibiotics, and the rates ranged from 7.4 to 51.7% [19, 21, 22, 27, 28, 31, 33–36, 38, 39, 41, 44, 46] and 14.6 to 44.8% [19, 21, 22, 28, 31, 33–36, 38, 39, 41, 44], respectively. This was followed by gentamicin (*n* = 8 studies; range: 6.6–22.3%) [19, 21, 22, 27, 31, 33, 34, 38, 44, 46], ampicillin (*n* = 6 studies; range: 6.0–29.2%) [27, 29, 31, 34, 44, 46], cefuroxime (*n* = 6 studies; range: 5.4–18.4%) [21, 27, 35, 36, 39, 41], ciprofloxacin (*n* = 6 studies; range: 7.8–17.4%) [21, 22, 27, 28, 33, 36], and amoxicillin-clavulanate (*n* = 6 studies; range: 8.8–13.4%) [22, 33, 35, 36, 41, 46]. Other antibiotic used include ampicillin-cloxacillin combination (*n* = 3 studies; range: 6–17.0%) [31, 33, 34, 44] and amoxicillin (*n* = 3 studies; range: 24.1–36.5%) [27–29]. Overall, only seven studies described antibiotics used based on the access, watch, and reserve (AWaRe) classification. The access group was the most commonly used antibiotics and ranged between 46.3 and 97.9% [20, 21, 32, 34, 45, 46], while the watch and reserve group accounted for 1.8–53.5% [20, 21, 32, 34, 45, 46] and 0.0–5.0% [20, 21, 32, 34, 45, 46], respectively.

#### Quality Indicators for Antibiotic Prescribing Among Hospitalized Patients

Eight studies describe the documentation of the reasons for antibiotic prescribing in patient notes [20, 27, 33, 34, 36, 38–40]. The results indicated that the rate of documentation ranged between 37.3 and 100%. The documentation of dates for stop/review ranged from 19.6 to 100% (*n* = 5 studies) [20, 33, 36, 39, 40] while taking specimen for microbiology culture ranged between 2.7 and 25% (*n* = 3 studies) [19, 20, 27]. The quality of antibiotic prescribing varied with the prevalence of prolonged surgical antimicrobial prophylaxis (administration for more than 24 h) ranging from 66.7 to 100% (*n* = 14 studies) [19–22, 30, 31, 33, 34, 36, 39–41, 45, 46]. One study reported that 6.2% of hospitalized patients with two or more antibiotics had redundant antibiotic prescriptions [22]. Table 3 shows the quality indicators of antibiotic prescribed among hospitalized patients.

**Table 3:** Quality indicators of antibiotic prescribed among hospitalized patients

S. no.	Author and year	Duration of SAP (%)	Document reason for antibiotic use in note (%)	Culture sample taken (%)	Document Stop/review dates in note (%)	Redundant antibiotic prescription (%)	Percentage of parenteral antibiotic (%)	Percentage of oral antibiotic (%)
1	Usman (2020) [22]	More than 1 day: 100	NA	NA	NA	6.2	55.7	44.3
2	Aboderin et al. (2021) [21]	More than 1 day: 99 More than 2 days: 94.8	NA	NA	NA	NA	89.9	19.9
3	Afriyie et al. (2020) [25]	NA	57.6–100	NA	29.1–100	NA	NA	NA
5	Amponsah et al. (2021) [27]	NA	88.1	2.7	19.6	NA	NA	NA
6	Ashour et al. (2022) [20]	More than 1 day: 98.5	100	25.0	NA	NA	98.6	NA
7	Bediako-Bowan et al. (2019) [19]	More than 1 day: 88.4	NA	4.0	NA	NA	54.0	46.0
8	Bunduki et al. (2021) [28]	NA	NA	NA	NA	NA	NA	NA
9	Nsofor et al. (2016) [29]	NA	NA	NA	NA	NA	NA	NA
10	Fentie et al. (2022) [30]	More than 1 day: 82.6	NA	NA	NA	NA	90.2	NA
11	Horumpende et al. (2020) [31]	More than 1 day: 89	NA	NA	NA	NA	NA	NA
12	Kamita et al. (2022) [32]	NA	NA	NA	NA	NA	54.0	20.8
13	Fowotade et al. (2020) [33]	More than 1 day: 100	92.4		21.3	NA	69.9	NA
14	Kiggundu et al. (2022) [34]	More than 1 day: 98.4	80.1	NA	NA	NA	88.0	11.0
15	Labi et al. (2018) [35]	NA	NA	NA	NA	NA	59.9	18.8
16	Labi et al. (2021) [36]	More than 1 day: 74.7	48.2	NA	46.7	NA	66.0	NA
17	Labi et al. (2018) [37]	NA	NA	NA	NA	NA	83.5	16.5
18	Momanyi et al. (2019) [38]	NA	37.3	NA	NA	NA	NA	NA
19	Nnadozie et al. (2021) [39]	More than 1 day: 96	97.5	NA	100	NA	63.9	36.1
20	Oduyebo et al. (2017) [40]	More than 1 day: 95	61.8	NA	27.8	NA	NA	NA
21	Ogunleye et al. (2022) [41]	More than 1 day: 76.2	NA	NA	NA	NA	83.1	NA
22	Okoth et al. (2018) [42]	NA	NA	NA	NA	NA	65.8	32.4
23	Omulo et al. (2022) [43]	NA	NA	NA	NA	NA	NA	NA
24	Seni et al. (2020) [44]	NA	NA	NA	NA	NA	NA	NA
25	Skosana et al. (2021) [45]	More than 1 day: 73.2	NA	NA	NA	NA	64.7	35.3
26	Skosana et al. (2021) [46]	More than 1 day: 66.7	NA	NA	NA	NA	76.7	23.3
27	Umeokonkwo et al. (2019) [47]	NA	97.5–100	NA	98.1–100	NA	64.3	35.7
28	Manga et al. (2021) [48]	NA	NA	NA	NA	NA	71.6	NA

*NA*, not applicable

## Discussion

This systematic review evaluated the prevalence, indication, and types of antibiotics used among hospitalized patients in Africa, as well as the quality indicators of antibiotic prescribing. The study found that there are limited studies that reported the prevalence of antibiotics used among hospitalized patients, particularly in the central and North African regions, where there was paucity of studies. The studies used different protocols including the World Health Organization protocol, global point prevalence survey protocol, and the European Centre for Disease Prevention and Control protocol to conduct the studies reflecting absence of an African protocol for conducting point prevalence of antibiotic use in African hospitals. Most of the included studies included were found to have good quality. The results showed that the prevalence of antibiotic use in inpatient settings in Africa is higher than the prevalence reported in Europe (30.5%) [17] and the USA (49.9%) [18]. This could be explained by the lack of adherence to antibiotic prescribing guidelines among prescribers [49, 50], inadequate knowledge of antibiotic prescribing among prescribers, and the misuse of antibiotics for the management of viral infections [51, 52]. The high rate of antibiotics used in inpatient settings in Africa highlights the need for antimicrobial stewardship program to promote rational use of antibiotics. ICU had the highest prevalence of antibiotic use, similar to the finding of the global point prevalence survey of 53 countries [53]. This was followed by pediatric medical, neonatal, and pediatric and adult surgical wards. This finding indicates the inpatient wards that should be prioritized for the implementation of antimicrobial stewardship program.

The current study also found that the most common indication for antibiotic use among inpatients in Africa was community-acquired infections. This is consistent with the finding in Europe [17], the USA [18], and the global PPS of antimicrobial use [53]. This result indicates the need to promote infection control and prevention strategies among the public to reduce the burden of community-acquired infections and eventually reduce the use of antibiotics. Surgical antimicrobial prophylaxis was the second most common indication for antibiotic use in African inpatient settings. This is not in agreement with the result of the global PPS where hospital-acquired infection is the second most common indication. It is important to note that about two-thirds to 100% of surgical antibiotic prophylaxis was prolonged beyond 24 h. In a similar study, more than half of the surgical antimicrobial prophylaxis prescriptions were prolonged beyond 24 h [17]. Excessive use of surgical antimicrobial prophylaxis contributes the emergence and spread of antimicrobial resistance. This result confirms the findings of previous studies that have demonstrated excessive use of surgical antimicrobial prophylaxis [22, 54]. Prolonged use of surgical antimicrobial prophylaxis is attributed to lack of knowledge among prescribers [55] and the use of antibiotics to augment suboptimal infection control and prevention practices. Therefore, surgical antimicrobial prophylaxis represents an important priority for the implementation of antimicrobial stewardship program in Africa. Previous studies have demonstrated the effectiveness of antimicrobial stewardship in improving the use of surgical antimicrobial prophylaxis and improving patient outcomes [12]. The results also showed that a considerable amount of antibiotics are used for hospital-acquired infections. High rate of hospital-acquired infections is attributed to poor infection control and prevention practices due to lack of training, lack of infrastructure, and high workload among healthcare workers in Africa [56, 57]. Therefore, infection prevention and control strategies including training of healthcare workers and promoting hand hygiene practices are recommended to reduce the burden of healthcare-associated infections and subsequently reduce antibiotic use in inpatient settings.

Ceftriaxone, metronidazole, gentamicin, ampicillin, cefuroxime, and ciprofloxacin were the most common antibiotics used among hospitalized patients in Africa. This was not consistent with the finding in Europe where beta-lactam plus beta-lactamase inhibitor combinations including amoxicillin-clavulanate and piperacillin-tazobactam; third-generation cephalosporins and fluoroquinolones were the most common antibiotics used in acute care hospitals [17]. In China, third-generation cephalosporin, fluoroquinolones, and metronidazole were the most common antibiotics used among hospitalized patients [58]. These variations are attributed to the differences in the burden of infectious diseases and the difference in antibiotic resistance pattern between the countries. In addition, high rate of ceftriaxone, metronidazole, gentamicin, and ampicillin usage could be attributed to the fact that they are relatively cheaper and have better safety profile than the beta-lactam beta-lactamase inhibitor combinations and fluoroquinolones. The high rate of ceftriaxone and ciprofloxacin usage in Africa is another important target for antimicrobial stewardship interventions. This is because these antibiotics are associated with increased risk of *Clostridium difficile* infection and the emergence of multidrug-resistant pathogens such as extended-spectrum beta-lactamase producing *Enterobacteriaceae*.

Most of the antibiotics used in Africa are in the access group while a considerable percentage of antibiotics belong to the watch group. However, the access group accounts for less than 60% of the antibiotics in most of the studies while the watch group accounted for more than 40% of the antibiotics in most of the studies. A previous study revealed that low-income countries had the highest access (62.8%), lowest watch 36.0%), and no reserve antibiotic prescription among adults compared to the other income groups [59]. The results of the current study imply that the antibiotics in the watch group were overused and those in the access group were underused. Therefore, interventions to promote more usage of antibiotics in the access group are recommended. Antibiotics in the watch group have higher potential for antibiotic resistance compared to those in the access group [60]. In addition, antibiotics in the reserve group are used for the treatment of multidrug-resistant infections. The low usage of the reserve antibiotics in Africa may be attributed to the non-availability of the antibiotics [59], and in some cases, the expensive cost of these life-saving medications may limit their use for those who pay for health services out-of-pocket. Therefore, interventions that promote accessibility, affordability, and availability of reserve antibiotics are recommended.

The general principle of antibiotic use requires taking specimen for microbiology culture and sensitive to guide definitive antibiotic therapy and minimize the risk of antibiotic resistance. The current study found that only one-quarter of patients receiving antibiotic therapy had specimen taken for culture. This shows that there is a major gap in the management of infectious diseases in Africa and highlights the need to strengthen laboratory capacity through diagnostic stewardship. The documentation of the reason(s) for antibiotic prescription was observed in most of the cases, although there is still room for improvement. The results also revealed that less than one-third of patients receiving antibiotics in Africa had a review/stop date documented in their case notes. The implication of this finding is the tendency to use antibiotics inappropriately and excessively. There was also report of redundant antibiotic combinations among inpatients in African hospital. These findings highlight some important opportunities for hospital pharmacists across Africa to participate in antimicrobial stewardship program. Therefore, training of hospital pharmacists and pharmacy students on antimicrobial stewardship is recommended [11, 61, 62].

The COVID-19 pandemic has caused significant disruption in healthcare systems across the world, and Africa is no exemption. The pandemic has affected both antimicrobial stewardship and infection control and prevention programs across the globe. Available evidence has shown that the pandemic has increased the rate of multidrug-resistant Gram-positive and Gram-negative pathogens [63]. There is paucity of data describing the impact of the pandemic on the prevalence and types of antibiotics prescribed among inpatients in Africa. Therefore, future studies should assess the impact of COVID-19 pandemic on antibiotic prescribing among inpatients in Africa. This study has a number of limitations including selection bias due to scarcity of point prevalence studies from Central African and North African regions. In addition, the exclusion of studies published in languages other than English language may have excluded relevant articles. Therefore, the findings may not be easily generalizable to the entire continent. Secondly, there was heterogeneity in the reporting of the prevalence as only a few studies reported the 95% confidence interval. This made it difficult to perform a quantitative analysis of the results. Therefore, a standardized protocol for conducting and reporting point prevalence survey of antibiotic use among inpatients in Africa is required to facilitate the performance of meta-analysis in the future. Despite these limitations, the current review provide some insights into the prevalence, indications, and types of antibiotics used among hospitalized patients in Africa as well as the quality indicators of antibiotic prescribing.

## Conclusion

The prevalence of antibiotic use among hospitalized patients in Africa is relatively high compared to Europe and the USA. The prevalence of antibiotic use was higher in adult intensive care unit and pediatric medical and neonatal wards compared to other wards. Antibiotics were most commonly used for community-acquired infections, followed by surgical antibiotic prophylaxis where more than two-thirds of the prescriptions was prolonged beyond 24 h. Broad spectrum antibiotics such as ceftriaxone, gentamicin, and fluoroquinolones were among the most common antibiotics prescribed among inpatients in Africa. Antimicrobial stewardship interventions are recommended, particularly in the surgical, ICU, and pediatric wards, to improve quality use of antibiotics in African hospitals and prevent antibiotic resistance.
